# Adolescents’ mental health and well-being in light of their substance use and the presence of special education needs

**DOI:** 10.1192/j.eurpsy.2024.714

**Published:** 2024-08-27

**Authors:** R. Dudok, B. Pikó

**Affiliations:** ^1^ Doctoral School of Education; ^2^Department of Behavioral Sciences, University of Szeged, Szeged, Hungary

## Abstract

**Introduction:**

Promoting mental health during adolescence is an essential health education objective and a crucial time for the formation of healthy mindset and behaviors. During this period, individuals are more likely to engage in health risk behaviors that can contribute to mental health problems that manifest in later adulthood. It has been demonstrated that optimal psychological health and the quality and application of students’ emotional and social skills may prevent and reduce the onset of risky health behaviors, such as subtance abuse. Students with specific learning difficulty (SpLD) are at higher risk to develop problem behaviors and they require special attention for promoting their mental health.

**Objectives:**

The aim of the present study is to investigate mental health and well-being, and health behaviors as well as substance use in a sample of adolescents including those with SpLD, using the SDQ ‘Strenghts and Difficulties Questionnaire’, a widely utilized instrument for the multidimensional assessment of mental health in children and adolescents.

**Methods:**

Our study included 276 school-aged children (mean age: 13.57 years; SD: 1.81; boys: 54.7%), 143 of whom had SpLD. We utilized a self-administered, anonymous questionnaire that included the Adolescent Psychological Well-Being Questionnaire, the Life Satisfaction Scale, and the WHO Well-Being Questionnaire. Peer support, individual internal psychological resources, and health risk behaviors were also assessed.

**Results:**

The statistical analyses revealed a number of noteworthy differences. First, the SDQ scores of smoking and drinking adolescents were substantially different from those of their peers on the dimensions of emotional symptoms, conduct problems, and hyperactivity in the case of smoking (p<.05), and on the dimensions of hyperactivity and prosocial behavior in the case of drinking (p<.05). On the other hand, significant differences were found between boys and girls, particularly in the domains of prosocial and affective symptoms (p<.05). Individuals with SpLD exhibited distinct patterns, particularly in the domains of emotional symptoms and peer relationship problems (p<.05). Furthermore, all of the investigated components of mental well-being had significant negative correlations with the SDQ dimensions of emotional symptoms, conduct problems, hyperactivity, and peer relationship problems, whereas the dimension of prosocial behavior showed a significant positive correlation (p<.05).

**Conclusions:**

Our findings support differences in mental health domains according to the adolescents’ substance using status or the presence of SpLD. The results of this study may contribute to the development of health promotion programs and intervention strategies as well as draw attention to the unique challenges faced by children with special education needs.

**Disclosure of Interest:**

None Declared

